# Non-Invasive Screening for Alzheimer’s Disease by Sensing Salivary Sugar Using *Drosophila* Cells Expressing Gustatory Receptor (Gr5a) Immobilized on an Extended Gate Ion-Sensitive Field-Effect Transistor (EG-ISFET) Biosensor

**DOI:** 10.1371/journal.pone.0117810

**Published:** 2015-02-25

**Authors:** Hui-Chong Lau, In-Kyu Lee, Pan-Woo Ko, Ho-Won Lee, Jeung-Soo Huh, Won-Ju Cho, Jeong-Ok Lim

**Affiliations:** 1 Biomedical Research Institute, Department of Biomedical Science, Kyungpook National University, Daegu, Korea; 2 Department of Electronic Materials Engineering, Kwangwoon University, Seoul, Korea; 3 Daegu Medical Center, Daegu, Korea; 4 Department of Neurology, Kyungpook National University Hospital Medical Centre, Chilgok, Korea; 5 Department of Materials Science and Metallurgical Engineering, Kyungpook National University, Daegu, Korea; Virginia Commonwealth University, UNITED STATES

## Abstract

Body fluids are often used as specimens for medical diagnosis. With the advent of advanced analytical techniques in biotechnology, the diagnostic potential of saliva has been the focus of many studies. We recently reported the presence of excess salivary sugars, in patients with Alzheimer’s disease (AD). In the present study, we developed a highly sensitive, cell-based biosensor to detect trehalose levels in patient saliva. The developed biosensor relies on the overexpression of sugar sensitive gustatory receptors (Gr5a) in *Drosophila* cells to detect the salivary trehalose. The cell-based biosensor was built on the foundation of an improved extended gate ion-sensitive field-effect transistor (EG-ISFET). Using an EG-ISFET, instead of a traditional ion-sensitive field-effect transistor (ISFET), resulted in an increase in the sensitivity and reliability of detection. The biosensor was designed with the gate terminals segregated from the conventional ISFET device. This design allows the construction of an independent reference and sensing region for simultaneous and accurate measurements of samples from controls and patients respectively. To investigate the efficacy of the cell-based biosensor for AD screening, we collected 20 saliva samples from each of the following groups: participants diagnosed with AD, participants diagnosed with Parkinson’s disease (PD), and a control group composed of healthy individuals. We then studied the response generated from the interaction of the salivary trehalose of the saliva samples and the Gr5a in the immobilized cells on an EG-ISFET sensor. The cell-based biosensor significantly distinguished salivary sugar, trehalose of the AD group from the PD and control groups. Based on these findings, we propose that salivary trehalose, might be a potential biomarker for AD and could be detected using our cell-based EG-ISFET biosensor. The cell-based EG-ISFET biosensor provides a sensitive and direct approach for salivary sugar detection and may be used in the future as a screening method for AD.

## Introduction

To date, most biomarker studies on Alzheimer’s disease (AD) have focused on the use of cerebrospinal fluid (CSF) and blood plasma as diagnostic specimens [1–7]. Despite the credited outcomes obtained from these studies, the invasive nature of obtaining CSF and plasma has underscored the need to search for an easy way to obtain a simple specimen for biomarker studies. Among all specimens, saliva is one of the simplest and easily accessible non-invasive body fluids. In the past, the use of saliva for diagnostics was often focused on periodontal diseases and oral health monitoring [8–10]. Recently, with the advent of advanced and improved biotechnological methods, much attention has been given to saliva as a useful body fluid for biomarker detection. Saliva specimens have been used in a wide range of applications and are considered useful not solely for their roles in food digestion or anti-bacterial properties. The complex and distinctive composition of salivary compounds fostered the exploration of their properties and potential uses in biomedical applications [10–12]. Consequently, if these properties of the salivary compounds present disease dependence, they may be used as alternative potential biomarkers for these diseases.

The prospects of using saliva as a specimen to diagnose diseases have raised interest among scientists following the transcriptomic and proteomic studies of salivary compounds. The extensive use of saliva as a medical diagnostic specimen has been reported for viral diseases, systemic diseases, and cancer [10,13–15]. These studies have suggested an association between salivary biomarkers and disease development. For instance, the levels of salivary electrolytes, such as sodium and calcium, were elevated in patients with cystic fibrosis, and HIV patients were found to have reduced salivary IgA levels [16–18]. Additionally, salivary microRNA and mtDNA have been found to be associated with tumors of parotid glands and head and neck cancer, respectively [19,20].

Saliva has especially gained attention in biomarker discovery for neurodegenerative diseases such as AD. Biomarkers for AD, like amyloid-β 42 (Aβ42), total-tau (t-tau), and phosphorylated-tau (p-tau) were previously reported as potential salivary biomarkers [13,21]. However, the source of these salivary protein biomarkers remained unknown. It has been suggested that these AD salivary biomarkers could be secreted from nerves into salivary glands due to their close proximity to the central nervous system [21]. Alternatively, salivary proteins could also be derived from ultrafiltration of blood at intracellular junctions [22]. Proteins could additionally be secreted into the saliva through diffuse and active transport [22,23]. Moreover, AD-related genes were found to be expressed in epithelial cells, suggesting yet another alternative source for salivary AD biomarkers [24–26].

In this study, we examined the reliability and practical usefulness of saliva as a specimen for the diagnosis of AD. Detection of Aβ42, t-tau, and p-tau was carried out to determine the sensitivity of salivary AD biomarker levels in AD patients. Because PD is another type of neurodegenerative disease with high prevalence, we included patients diagnosed with PD as one of the patient groups. Furthermore, PD has distinct mechanisms, indicators, and symptoms that are different from AD. These features make PD patients a suitable patient group that can be distinguished from AD to determine the sensitivity and selectivity of these biomarkers. To our knowledge, there were only two reports on salivary biomarkers for AD [13,21]. In contrast, several studies discussed the use of CSF and plasma as sources for AD biomarkers [2–4,6,27]. Therefore, further studies of potential salivary AD biomarkers are necessary to validate their use.

We also developed a screening method for AD using a biosensor. Our preliminary study identified several types of salivary sugars in AD that lead us to investigate their use as biomarkers for AD [28]. In light of these findings, we developed a highly sensitive, salivary-based screening method for neurodegenerative diseases, AD in particular, by using *Drosophila* cells and an extended gate ion-sensitive field-effect transistor (EG-ISFET) sensor. The cell-based EG-ISFET sensor utilizes gustatory receptors (Gr5a) expressed abundantly in *Drosophila* cells to detect trehalose. Gr5a is found to be expressed abundantly in the gustatory neurons in the sensory hairs of *Drosophila*. This receptor responds specifically to sugar, especially trehalose. The EG-ISFET sensor we used in this study is a type of chemical transducer derived from the metal oxide semiconductor field-effect transistor (MOSFET) described in various types of sensing applications [29–31]. The EG-ISFET sensor measures the concentration of ions in a solution based on the interaction between ions and a metal oxide membrane. Variations in ion concentration cause changes in the threshold voltage, which, in turn, leads to difference in the current. Cells expressing Gr5a were immobilized on the EG-ISFET sensor and the sensor was used to screen for the salivary sugar, trehalose in AD, PD, and control groups. These findings, obtained using the cell-based EG-ISFET biosensor, present a new frontier in biosensing-based tools for detection of AD by using saliva samples. This type of biosensor may also be used as a quick alternative screening method for future diagnostic purposes.

## Materials and Methods

### Collection protocol for human saliva samples

A total of 60 unstimulated human saliva samples were obtained from participants recruited from the Kyungpook National University Hospital. Each group, namely, healthy individuals, AD patients, and PD patients, was composed of 20 participants. All participants had previously undergone a series of clinical and neuropsychological examinations by physicians and neuropsychologists. In the control group, participants were at least 50 years of age or older, in good general health, and had no history of neurological, psychiatric or major medical diagnosis that could contribute significantly to cognitive impairment or dementia.

Neuropsychological tests such as the Mini Mental State Examination (MMSE) and the Clinical Dementia Rating-Sum of Boxes (CDR-SOB) scores were consistently determined to distinguish between those diagnosed with AD from patients diagnosed with PD and the healthy individuals from the control group. In this study, the AD group had an average MMSE score of 18.15 ± 5.4, whereas the scores for the PD and the control groups were 22.45 ± 5.46 and 28.7 ± 1.11, respectively. The CDR-SOB rating, for the control group was 0.23 ± 0.25, whereas the PD and AD groups had scored of 2.6 ± 2.94 and 6.25 ± 2.67, respectively. The demographic and clinical data for these samples are shown in Table 1.

**Table 1:** Demographic and clinical data of the study participants.

**Characteristic**	**AD N (20)**	**PD N (20)**	**Control N (20)**	***p*-value**
**Age, (Y)**	72.5 ± 7.68	73 ± 8.07	66.1 ± 7.79	-
**Male sex-no (%)**	40	45	25	-
**Smoker (%)**	15	10	15	-
**Ex-smoker (%)**	5	20	5	-
**MMSE**	18.15 ± 5.4	22.45 ± 5.46	28.7 ± 1.11	≤ 0.05
**CDR-SOB**	6.25 ± 2.67	2.6 ± 2.94	0.23 ± 0.25	≤ 0.05
**H-Y**	NA	2.31 ± 0.58	NA	

Values are represented as mean ± SD. MMSE scores range from 0 (severe impairment) to 30 (no impairment). A score of higher than 27 was considered normal. CDR-SOB scores range from 0 (cognitive normality) to 18 (maximal cognitive impairment). Hoehn and Yahr (H-Y) scores range from stage 1 (mild) to stage 5 (maximal disabling). The *p*-value that evaluates statistical significance between these variables was computed using the one-way ANOVA.

Participants who met the criteria of selection were then requested to fast for at least 4 h prior to spitting 3 mL of their saliva for sample collection. The unstimulated saliva samples were centrifuged at 1000 × *g* for 15 min to remove debris. A proteases inhibitor cocktail (Promega Corporation, Madison, WI, USA) was added to each supernatant per the manufacturer’s recommendations to prevent protein degradation. Samples were stored at -80°C until use.

### Ethics Statement

The collection method used to obtain saliva samples was reviewed and approved by the Institutional Review Board (IRB) of the Kyungpook National University Hospital (IRB code KNUH_12–1002). Consent forms were obtained from all participants recruited for this study. To protect the patient information, the samples were assigned a specific code number and stripped of any personal information before being used in the study.

### ELISA quantification of salivary AD biomarkers

Aβ42, t-tau, and p-tau were quantified using human-specific enzyme-linked immunosorbent assay (ELISA) (Biosource International, Invitrogen, Carlsbad, CA, USA). The assays were performed according to the manufacturer’s instructions. Approximately, 50 μL of saliva sample was used to determine the levels of each biomarker in patient and control groups. In order to obtain reliable data, all experiments were conducted in duplicate. Salivary levels of the AD biomarkers were plotted using GraphPad Prism 5.00 (GraphPad Software, San Diego, CA, USA). The differences between each biomarker in the AD, PD, and the control groups were examined using the one-way ANOVA statistical approach. Results were considered significant when calculated *p*-values were ≤ 0.05.

### Expression of Gr5a in *Drosophila* S2 cells

Construction of the Gr5a expression vector was previously described by our group [32]. Briefly, the *Gr5a* gene was extracted from *Drosophila* and amplified using polymerase chain reaction (PCR). The amplified product was cloned into the pAc5.1/V5-His/lacZ plasmid DNA. The resulting plasmid was transfected together with the selection vector, pCoHygro, into *Drosophila* S2 cells with polyethylenimine (PEI). Transfected cells were incubated at 28°C overnight. After 3 to 4 days of incubation, the transfected cells were cultured in a selective medium containing 300 μg/mL hygromycin to select for stable cells expressing Gr5a. The selection was carried out for 4 weeks, and the resulting stable cells were used to further scale up expression. For the cell-based EG-ISFET study, the cells expressing Gr5a were used as trehalose-sensing cells, whereas untransfected *Drosophila* S2 cells grew under the same condition as the Gr5a expressing cells were used as control cells.

The stable cells expressing Gr5a were verified using a reverse transcriptase PCR (RT-PCR) protocol. The RT-PCR product was then sequenced to confirm the expression of Gr5a. Briefly, the total RNA was first extracted using RNeasy Mini Kit (Qiagen, Hilden, Germany) and amplified using a one-step RT-PCR kit (iNtRON Biotechnology, Seongnam, Korea) per both manufacturers’ recommendations. The amplification reactions were carried out in 20 μL reaction mixtures containing 0.5 μg of total RNA using a Bio-Rad thermal cycler (Bio-Rad Laboratories, Hercules, CA, US). The cycling conditions were as follows: 30 min at 45°C, followed by 5 min at 94°C and 35 cycles of 30 s at 94°C, 56°C for 30 s, and 1 min at 72°C. The following primers were used: Gr5a forward primer: CTGTTTTATTCCTCATCACTGGCC and the Gr5a reverse primer: GTCCATGTAACTCCAGCCGAAGGT. The PCR products were run on a 1% agarose gel and visualized under an UV transilluminator. The gel photo was captured using the Chemi Genius, Bio Imaging System (SynGene, Frederick, MD, USA). Next, the PCR products were purified and sequenced to confirm the expression of Gr5a. Sequence data were generated by Bioneer, Daejon, Korea.

### Development of the cell-based EG-ISFET biosensor

Construction of the EG-ISFET sensor was slightly different from a conventional ISFET device. The gate terminal, with an oxide membrane of the EG-ISFET, was segregated from the MOSFET part. Instead of fabricating the transistor in-house, we used a commercially available transistor (MC14007UBCP) as the MOSFET part. The fabrication process of the sensing and the reference regions of the EG-ISFET device were as follows. First, a 150-nm thick, titanium (Ti) layer was deposited on p-type Si (100) wafers with 300-nm thick thermal oxide using an electron-beam evaporator. The Ti layer aids in the transfer of the electrical potential changes of the surface of the sensing membrane surface to the Si wafer. Next, a post-deposition annealing (PDA) at 450°C for 30 min in ambient N_2_ gas was carried out using a furnace. The furnace also helped improve the electrical properties of the device. Then, a 25-nm thick, tin oxide (SnO_2_) film, which functions as the sensing membrane of the EG-ISFET, was deposited using radio-frequency (RF) magnetron sputtering. The deposition power was set to 50 W, the working pressure was set to 3 mTorr, and the Ar gas flow was set to 20 sccm. Finally, a reservoir to contain the sample solution was constructed on top of the gate terminal from poly(dimethylsiloxane) (PDMS).

### Measurement of sensitivity and chemical stability of EG-ISFET sensor

Sensitivity of SnO_2_ membrane of the device was determined by measuring the current-versus-voltage (*I-V*) curves using various pH buffer solutions (pH 3–11). Besides, chemical stability of EG-ISFET sensor device was determined based on the drift rate of sensing membrane at pH 7 buffer solution. Measurement of drift rate was carried out by determining the difference of gate voltage over time. Drift rate is an indicator of sensor device chemical stability for long term. The (*I-V*) curves and gate voltages were measured using a Hewlett-Packard 4156B High-Precision Semiconductor Parameter Analyzer (Hewlett-Packard Co., Palo Alto, CA, USA) and commercial Ag/AgCI reference electrode. Measurements were carried out in a dark box at room temperature to avoid interference with light and noise.

### Measurement of the current using the cell-based EG-ISFET biosensor

The selectivity and sensitivity of the fabricated EG-ISFET sensor was evaluated with various concentrations (0.001, 0.01, 1M) of trehalose. The cell-based EG-ISFET sensor was tested using human saliva samples. Briefly, 100 μL of cell suspension, at a density of 5 × 10^5^ cell/mL, was transferred into the reservoirs of both sensing and reference regions on the EG-ISFET sensor. The cell suspension was gently mixed with 30 μL of 100 μM phosphate buffered solution (PBS) and the stable current baseline was measured. Once a stable baseline was achieved, 30 μL of trehalose solution was added to both sensing and control regions and measured after each addition until the final concentration was reached. The stable cells expressing Gr5a were used as the sensing cells, whereas the untransfected S2 cells were used as a control to validate the currents obtained from the measurements. The change in the current at each concentration was evaluated for all measurements. At least two independent measurements were taken for each experiment.

Eighteen saliva samples from the AD, PD, and control groups were screened for salivary trehalose levels using the fabricated cell-based EG-ISFET sensor. The stable cells expressing Gr5a were used as the sensing cells on both reference and sensing regions of the EG-ISFET sensor. This setup allowed us to make a comparative measurement of the control and patient groups simultaneously. Saliva from the AD and PD groups were examined on the sensing region, whereas the control saliva samples were tested using the reference region. Additionally, samples from the AD, PD, and control groups were examined using the difference in the current generated from the sensing and control cells. This was performed to verify the response of overexpressed Gr5a cells to trehalose in the saliva samples and variation between the disease and control groups.

All response currents generated were measured using a Hewlett-Packard 4156B High-Precision Semiconductor Parameter Analyzer. The changes in the current from the EG-ISFET sensor were evaluated using the change of current generated from both the sensing and the reference regions of the EG-ISFET sensor. The stable saturated current response was determined to represent the detection of the response from both sensing and reference regions.

### Statistical tests

All data obtained from the ELISA assays and the EG-ISFET biosensor were analyzed using GraphPad Prism Software 5.00 (GraphPad Software). The median of duplicate experiments are reported. The differences between groups were determined using the Student t-test and one-way ANOVA. These values were considered significantly different with the calculated *p*-value was ≤ 0.05.

## Results and Discussion

The ELISA immunoassay results did not demonstrate a significant difference in the levels of the tested AD biomarkers between the patient and control groups. The AD biomarkers Aβ42, t-tau, and p-tau at the levels contained in saliva did not reflect AD progression. Salivary p-tau was found to be expressed higher in AD group than in the PD and control groups. (Figure A in S1 File). Although, there was no significant difference detected between these groups by using this method, our findings are similar to those previously published [21]. On the other hand, our study found no significant differences in the salivary t-tau levels of the AD, PD, and control groups (Figure A in S1 File).

The biomarker Aβ42 was not detected in the saliva samples used in this study. Salivary Aβ42 was first reported to be detected in the saliva samples in 2011 [13]. These authors found a small, but statistically significant, increase in the levels of salivary Aβ42 in patients diagnosed with a mild form of AD. Salivary levels of Aβ42 measured in their study were not significantly different from the PD and control groups. Another report, published later the same year, reported that Aβ42 was not detected in human saliva samples [21]. Again, our results are similar to Shi *et al.*, as we did not detect salivary Aβ42 in most of the analyzed samples. These discrepancies may be due to the low levels of salivary Aβ42 in the samples. In addition, Shi *et al.*, suggested that the hydrophobic and heterogeneous domains of Aβ42 may have had contributed to the low sensitivity [21].

It has been shown that monitoring the progression of AD has a higher success rate by using CSF AD markers [5,33]. AD biomarkers from CSF were reported to have higher confidence than plasma AD biomarkers. Initially, it was believed that plasma would be the body fluid of choice in the search for AD biomarkers and that it would replace CSF sampling. However, subsequent studies showed that plasma Aβ42 did not correlate with the accumulation of Aβ42 in the brain of AD patients when post-mortem examinations of both were carried out simultaneously [5,33]. As a result, it was suggested that plasma Aβ42 might not be associated with the metabolism of amyloid-β peptides in the brain [27]. Consequently, the low sensitivity of salivary AD biomarkers may also imply a weak correlation between salivary Aβ42 levels and the accumulation of amyloid-β in the brain. Therefore, this could explain why studies using immunoassays and existing salivary AD biomarkers were not able to detect a strong correlation to AD progression. Hence, biosensors could be used as viable alternative approach to study salivary compounds.

The cell-based EG-ISFET biosensor we developed is a novel screening approach for AD, as to our knowledge, no other study has reported the use of an EG-ISFET sensor for AD screening with saliva. The EG-ISFET sensor coupled with *Drosophilla* cells for trehalose detection in saliva was specifically designed for the screening of AD patients and the detector is more sensitive than optical sensors such as those used in surface plasmon resonance. Additionally, EG-ISFET sensors provide accurate measurements in real time by allowing direct measurement of the interactions between substrates and analytes with an observable change in current. Moreover, the sensor device for EG-ISFET is small and easily fabricated for portable usage.

**Fig 1:**
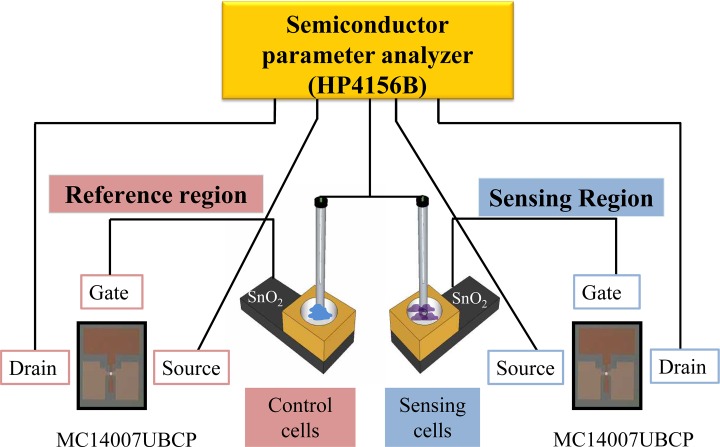
Top view image of the EG-ISFET sensor device. The EG-ISFET sensor consists of an independent reference and sensing region for simultaneous measurements of samples from patient and control groups. A commercially available transistor (MC14007UBCP) was used as the MOSFET part of the sensor. The gate terminal of both sensing and reference regions was segregated from the conventional ISFET device. A SnO_2_ membrane was deposited on the gate terminal of the EG-ISFET sensor and used as the sensing membrane. Response currents generated were measured using a Hewlett-Packard 4156B High-Precision Semiconductor Parameter Analyzer.

As an improvement on the design of the conventional ISFET, our biosensor makes use of the extensible gate terminal of MOSFET to allow construction of two independent components: one used for reference and the other used for sensing (Fig. 1). These components are essential to obtaining accurate measurements. Because of this unique design, the responses from the patient and control groups can be generated and observed concurrently.

Most of the studies for cell-based ISFET sensor report the use of mammalian cells, which are sensitive to temperature change, leading to a reduction in viability. In order to overcome this, we used *Drosophila* cells, which can be grown at 25°C to maintain the cellular activity. *Drosophila* cells expressing Gr5a were loosely and semi-adhered on a thin layer of SnO_2_ membrane deposited on the EG-ISFET gate terminal. This ensured that the changes due to reaction with the saliva from the AD, PD, and control groups could be measured.

**Fig 2:**
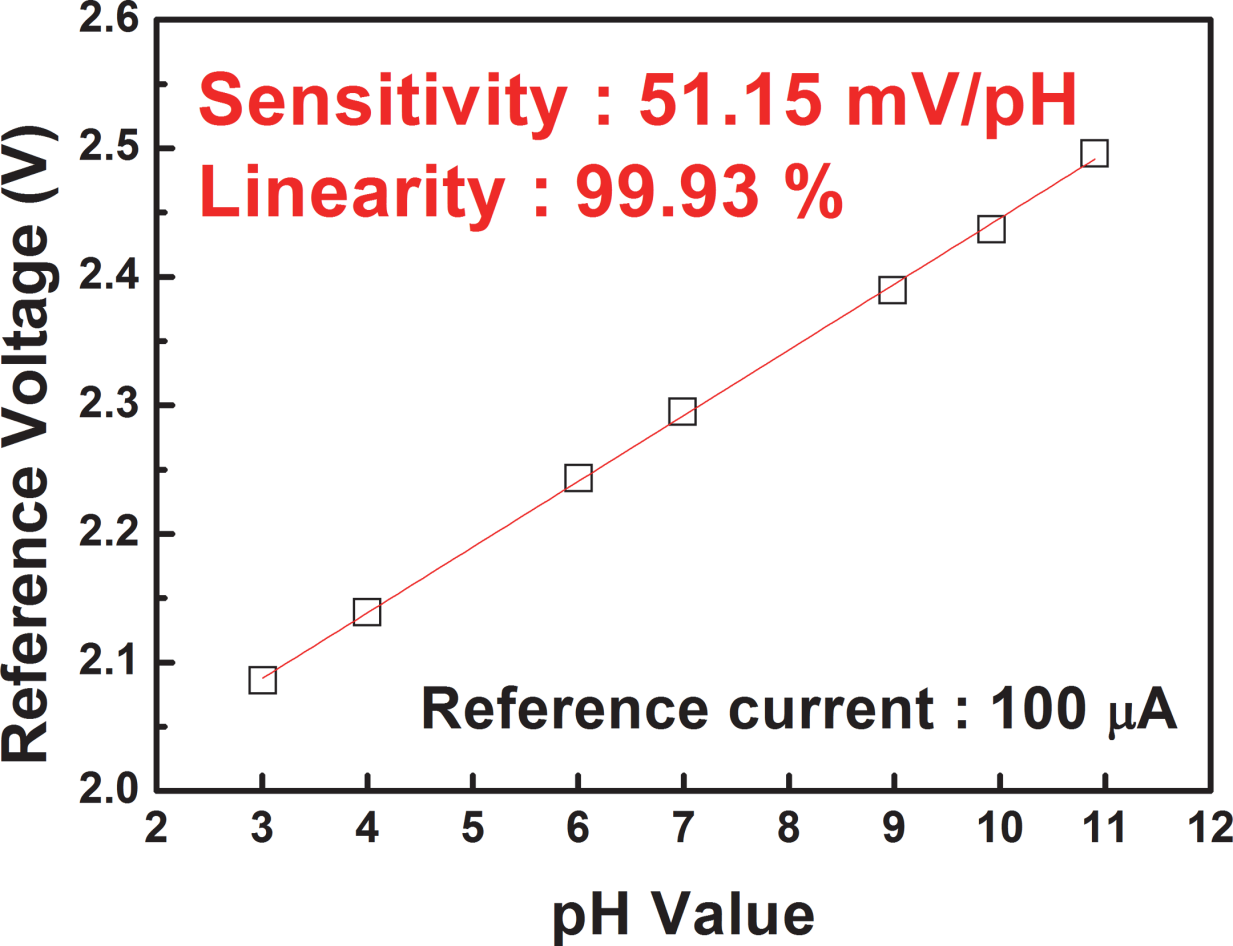
Effect of the pH on the sensitivity of SnO_2_ membrane. The reference voltage (V) is shown as a function of pH evaluated at a reference current of 100 μA.

**Fig 3:**
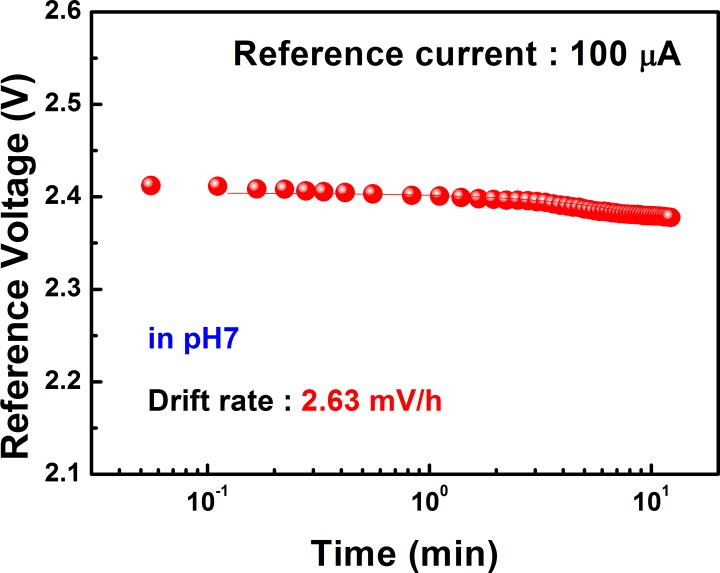
Drift rate of SnO_2_ membrane in a solution with pH 7. The reference voltage (V) is shown as a function of pH evaluated at a reference current of 100 μA.

Prior to performing the measurement using cells, we determined the pH sensitivity and chemical stability of the EG-ISFET sensor device. The EG-ISFET with the SnO_2_ membrane showed a pH sensitivity of 51.15 mV/pH and a linearity of 99.93% (Fig. 2). The pH sensitivity of the SnO_2_ sensing membrane we used in this study is very close to the Nernstian limit of 59 mV/pH. This suggests a high sensitivity of the EG-ISFET sensor. The drift rate of the membrane was found to be 2.63 mV/h indicates excellent stability of the sensor device (Fig. 3). We found that the pH sensitivity and stability of the SnO_2_ sensing membrane are important factors for the efficiency, stability, and sensitivity of the sensor. After this quality check, the cells can be applied and the sensor can be used as a cell-based biosensor. We have previously fabricated a cell-based ISFET sensor by using a silicon oxide-based membrane. This setup also showed good sensitivity [32]. However, the EG-ISFET sensor, fabricated using the SnO_2_ membrane described in this study, had a higher pH sensitivity and stability than the previous design. These results demonstrate that this improved EG-ISFET sensor is more efficient than the previous design.

In order to verify the stable expression of Gr5a in *Drosophila* cells, we also examined the expression of Gr5a using RT-PCR. The results showed that the stable cells expressed recombinant Gr5a abundantly compared to the endogenous expression in *Drosophila* S2 cells (Figure B in S1 File). The overexpression of Gr5a was demonstrated by the presence of a higher number of cDNA copies of the *Gr5a* gene in the stable cells than the endogenous *Gr5a* gene from S2 cells when the same amount of RNA was used as the initial template. These studies demonstrated that the sensing cells contained overexpressed Gr5a for trehalose sensing. Previously, we have optimized the detection condition using ISFET sensor and the required number of Gr5a expressed cells to detect the presence of trehalose [32]. We found that the reservoir of sensor device, approximately 1 cm × 1 cm, cells at 5 × 10^5^ cell/mL was optimum for detection. With the same size of reservoir sensor for EG-ISFET sensor, cells at 5 × 10^5^ cell/mL was able to obtain consistent detection. Based on our previous experiments, number of cells either higher or lower than the optimum cells number resulted in an inconsistent detection, poor signal to noise ratio, and off limit detection. As a result, we could only report the use of cells at one concentration for detection at this point of time.

**Fig 4:**
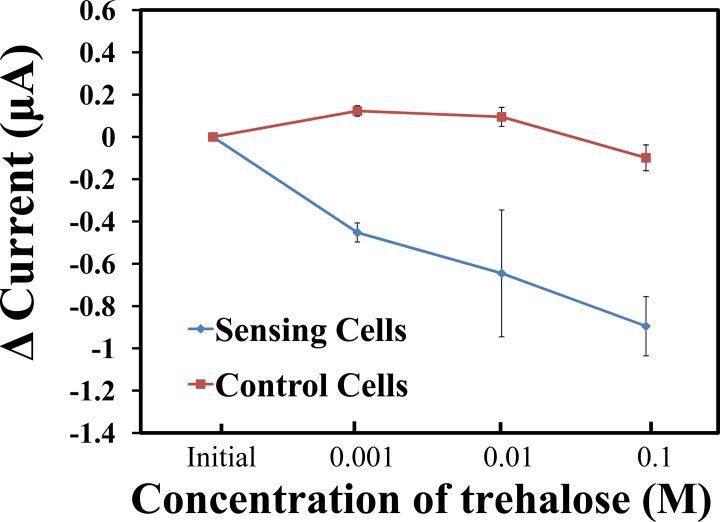
EG-ISFET biosensor response currents of cells reacting to trehalose. The change of current determined with the EG-ISFET sensor is represented as a function of trehalose concentration. The sensing cells depicted as closed red squares, whereas the control cells are shown as closed blue circles.

The response generated by the EG-ISFET sensor using these cells was first examined using different concentrations of trehalose. As the concentration of trehalose increased, the sensing region, which contains the sensing cells, had a steep decrease in the current compared to the control cells on the reference region (Fig. 4). The observed current reduction might be because more hydroxide ions are being detected by the sensing cells. An exchange of ions occurs upon the binding of the sugar molecules to the gustatory receptors of *Drosophila* cells. These, in turn, alter the number of hydroxide ions available, and subsequently, cause a shift in the threshold voltage to the right, resulting in a reduction of the generated current. A minimum change in the current was observed with the control cells lacking the overexpression of the gustatory receptor Gr5a. These results indicate that the sensing cells responded efficiently to trehalose up to 0.001 M.

**Fig 5:**
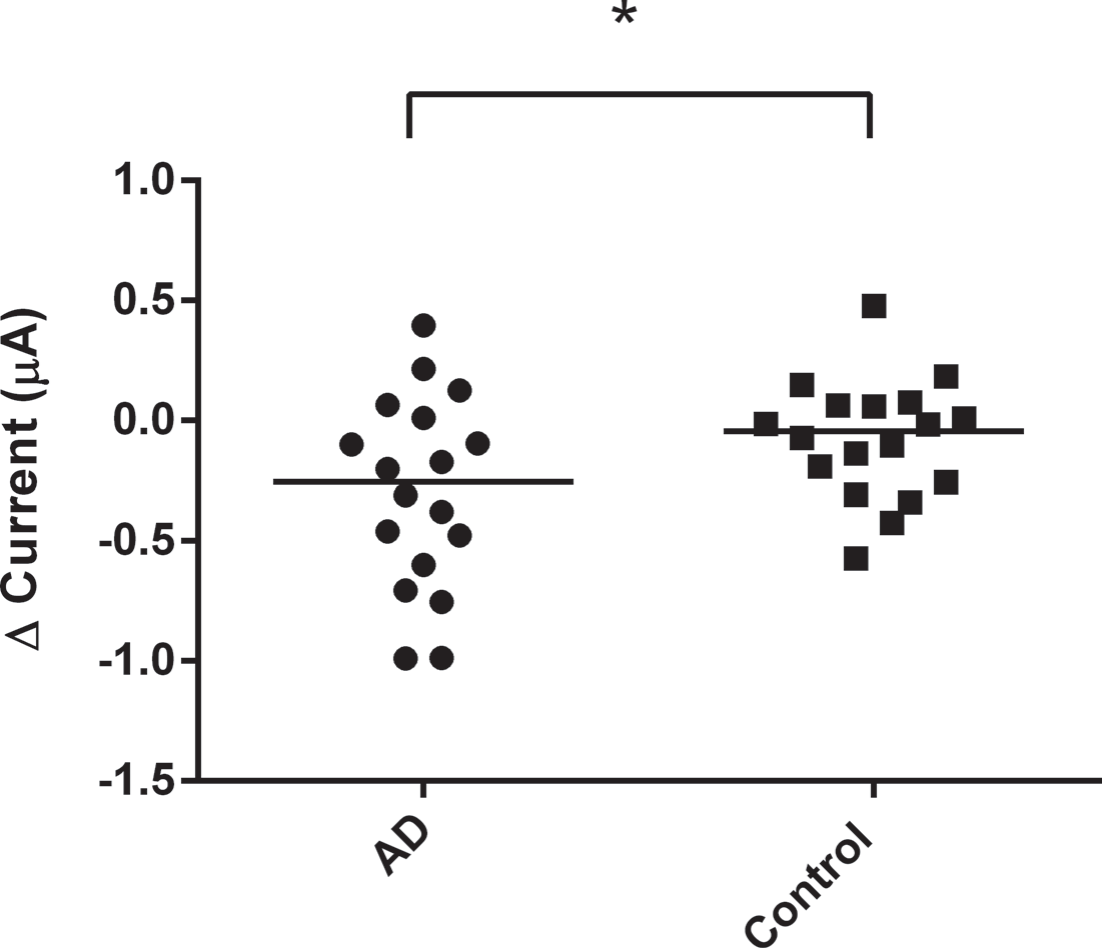
Change in EG-ISFET biosensor currents for AD and control saliva samples. The change of current generated from the AD and control groups. Data are presented as median. *p ≤ 0.05.

**Fig 6:**
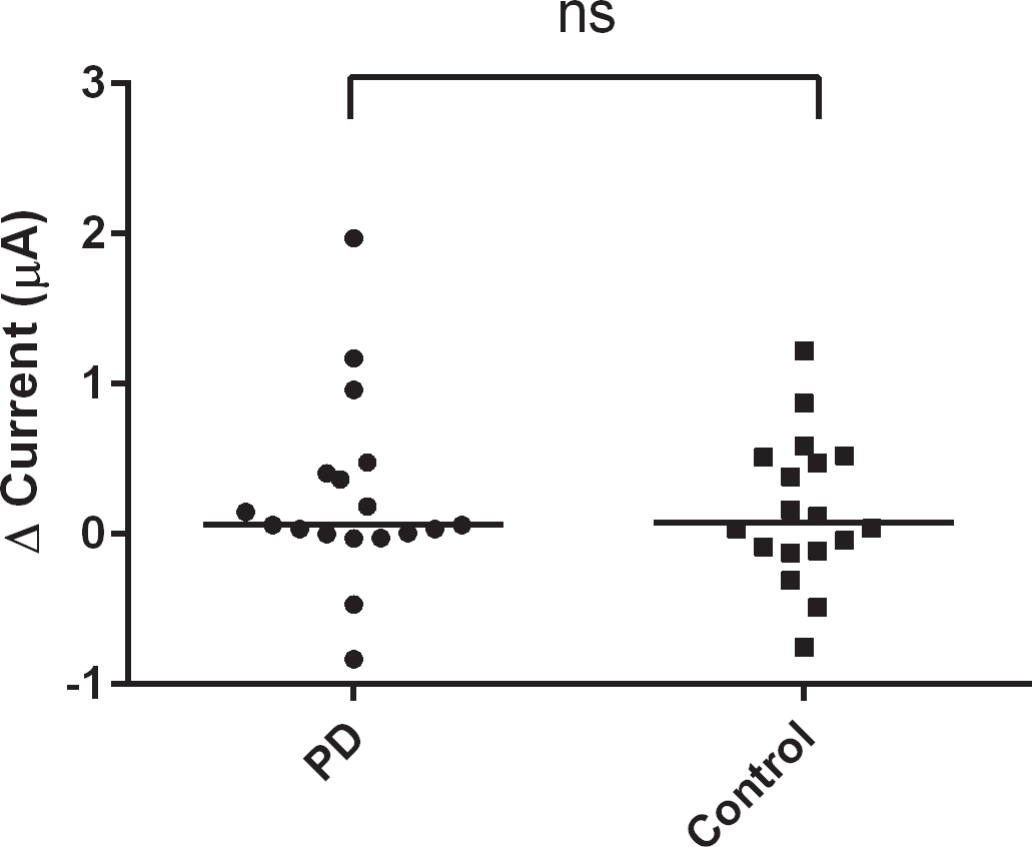
Change in EG-ISFET biosensor currents for PD and control saliva samples. The change of current generated from the PD and control groups. Data are presented as median. *p ≤ 0.05; ns = not significant

**Fig 7:**
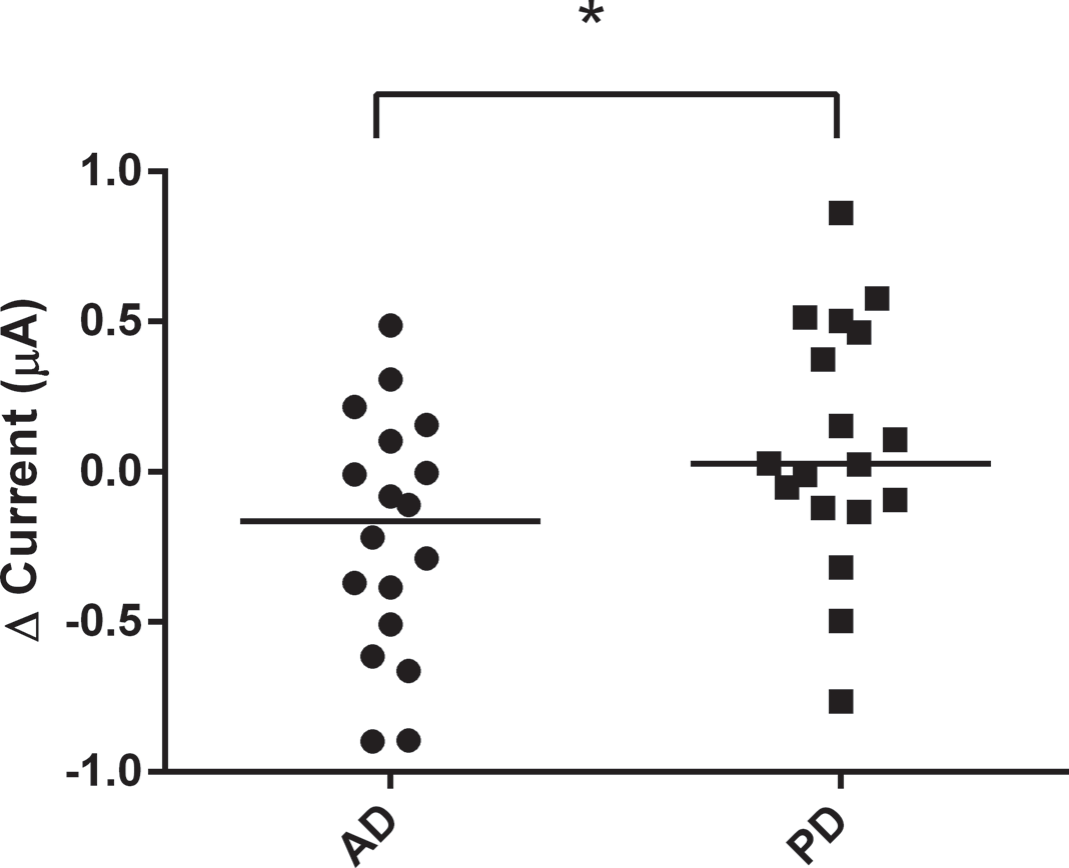
Change in EG-ISFET biosensor current for AD and PD saliva samples. The change of current generated from the AD and PD groups after the values were normalized to the values obtained from the control group of healthy individuals. Values are represented as median (left). *p ≤ 0.05.

After obtaining stable and reliable results using trehalose, the EG-ISFET biosensor was then used to detect the change of response to saliva samples collected from patient and control groups. The saliva samples of the control group resulted in a higher change in the current than the AD (Fig. 5) and PD groups (Fig. 6). The control group in Fig. 6 had a response current of 0.078 whereas the current for PD group was 0.016. Although the difference was small, the response current of control group was still higher than the PD group. After the values were normalized to the control group, the PD group was found to have higher change of current compared to the AD group (Fig. 7). We found a statistically significant difference between the AD and the other two groups.

We also carried out additional experiments to examine the differences in the generated currents of the sensing and control cells when identical saliva samples were used. The AD group was observed to have a lower current change compared to either the PD or the control groups (Figure C in S1 File). These results showed a similar pattern to the sensing cells. The increase in currents in the control group might be due to the generation of hydrogen ions upon binding of trehalose to the Gr5a expressed in the cells suggesting that salivary trehalose levels in the patient and control groups are different. Additionally, we also found that the sensing cells with overexpressed Gr5a receptors had higher change in current than the control cells in the reference region when we examined the response of saliva samples of both cells simultaneously (Figure D in S1 File). This suggests that the changes in current from the sensing region were due to the detection of trehalose instead of other compounds in the saliva.

Based on the detected variations in the current change, there is a high potential in the use of cell-based biosensors to screen for AD, based on the response of cells expressing Gr5a to trehalose. Therefore, salivary compounds like sugars could serve as potential biomarkers for AD. High salivary sugar levels were previously thought to be associated with the occurrence of diabetes mellitus [34,35]. Several reports have proposed an association between diabetes mellitus and progression of AD [36,37]. In addition, AD patients were also observed to have a high occurrence of diabetes mellitus [36,38–40]. These data provide strong support that high salivary sugar levels are correlated with AD development. Furthermore, this correlation can be established using the biosensor described in this study. Our study also found that while the PD group had lower salivary trehalose levels than the AD group, the levels were higher (although not significant) than those recorded in the samples from the control group. The control group showed lower salivary sugar levels than the patient groups. These results imply that the saliva of AD patients might have a higher sugar content than PD patients, whereas PD patients had an increased sugar content compared to the controls. Hence, salivary sugar levels of the AD group could be clearly distinguished from the control and PD groups by using a cell-based biosensor device.

We studied the correlation of salivary trehalose sugar levels with AD progression because sugar is one of the prominent biomolecules. Sugars play an important role in metabolism and are often associated with physiological and metabolic changes in the body. Although the source of salivary trehalose remains unknown, salivary sugars could possibly be derived from blood via several ultrafiltration, transcellular, and paracellular pathways, in light of the correlation between salivary and serum glucose [41]. Thus, sugar composition in the saliva could be associated with disease development, and the use of an EG-ISFET biosensor to detect a sugar-like component such as trehalose, could serve as an alternative diagnostic tool for AD.

## Conclusions

Our study describes a thorough investigation on the use of a non-invasive method using saliva samples as a screening method for AD. Based on the EG-ISFET sensor, we developed a cell-based biosensor and demonstrated that saliva samples from AD patients could be significantly distinguished from those of healthy individuals and PD patients. The cell-based EG-ISFET sensor we designed contains two components; one for response current generated from a sensing region and another for reference. The EG-ISFET sensor had a pH sensitivity of 51.15 mV/pH and drift rate of 2.63 mV/h, which were excellent for detection.

Gr5a overexpression in *Drosophila* cells bound to the sensing enabled the detection of up to 0.001 M of trehalose. The differences in the generated current in AD patients, PD patients, and samples from healthy individuals determined by the biosensor may be attributed to the differences in the salivary sugar levels of trehalose. Consequently, this may be a strong indication that AD patients have a higher salivary sugar content than healthy individuals or PD patients. In addition, the samples were obtained by a non-invasive method and could be an alternative, reliable specimen for the sensor-based screening approach to detect potential biomarkers. This, in turn, could help estimate the risk of developing AD. With the success of the *Drosophila* cell-based EG-ISFET sensor, fabrication of a mini ISFET device could accelerate screening processes and be more cost effective for screening larger populations. Additionally, with fabrication of a synthetic Gr5a receptor chip, personal screening for AD at home is possible. This could aid in an improved prognosis of the disease.

## Supporting Information

S1 File Figures A-D. Figure A. Salivary AD biomarkers for three different groups. Salivary t-tau (right) and p-tau (left) levels in AD patients, PD patients, and healthy individuals. Data are presented as mean ± SEM. Figure B. The gel picture shows the amplified PCR product. The size of the PCR product was found to be at the expected size. Line 1: 1-kb marker, Lane 2: S2 cells, Lane 3: stable cells expressing Gr5a. Figure C. Change of currents for AD, PD, and control groups using the EG-ISFET biosensor. The change of current generated from the sensing cells after it was normalized to the values obtained from control cells. Data are presented as median. Figure D. Change of currents for sensing and control cells using EG-ISFET biosensor. The change of current generated from the sensing and control cells from saliva samples. Data are presented as median.
(ZIP)
