# Supplementary figures and images for: Non-Invasive Screening for Alzheimer’s Disease by Sensing Salivary Sugar Using Drosophila Cells Expressing Gustatory Receptor (Gr5a) Immobilized on an Extended Gate Ion-Sensitive Field-Effect Transistor (EG-ISFET) Biosensor
Source: PLoS One. 2015 Feb 25;10(2):e0117810. doi: 10.1371/journal.pone.0117810 (PMC4340960; doi:10.1371/journal.pone.0117810)

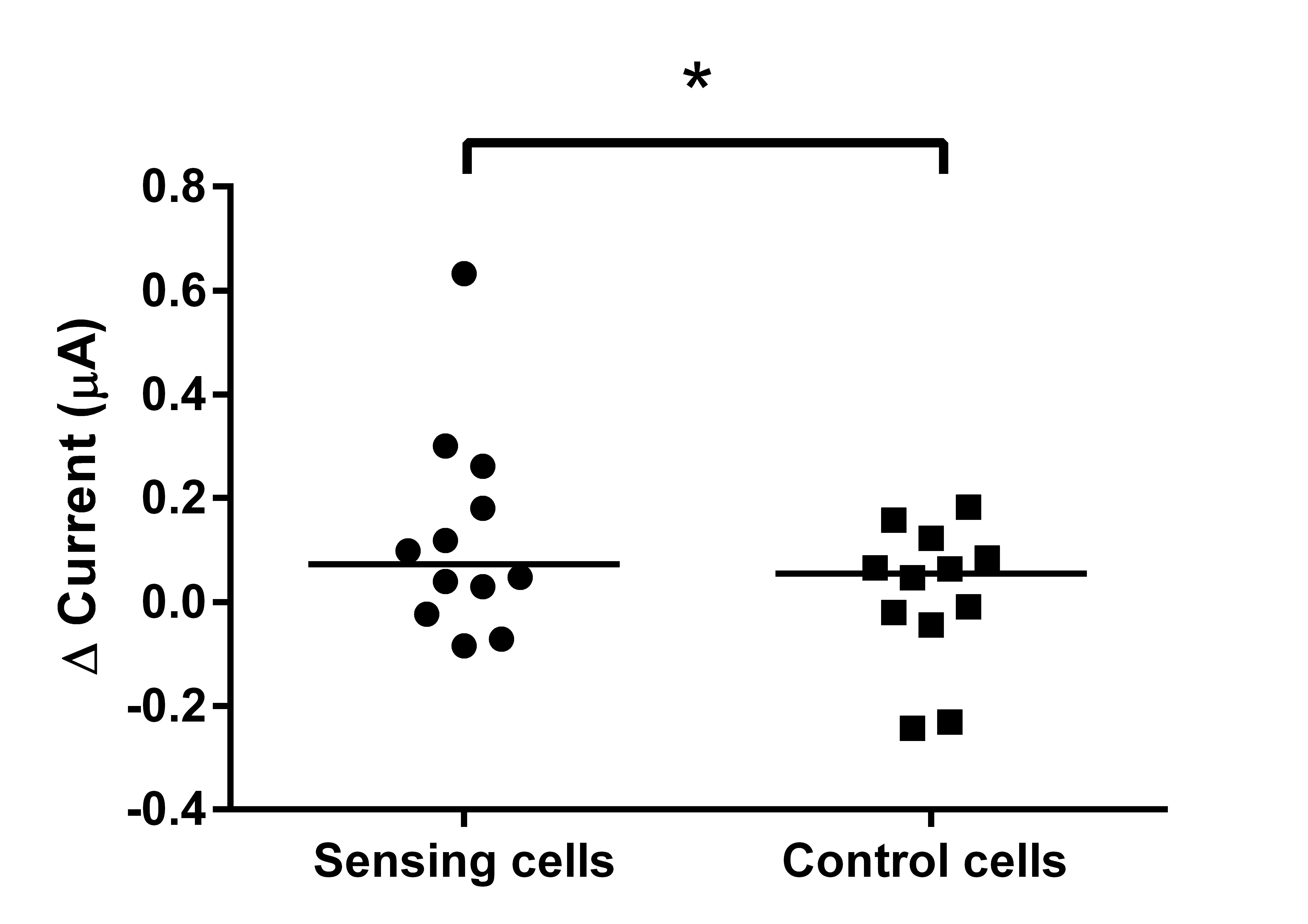

Supplement: S1 File — Figure A. Salivary AD biomarkers for three different groups. Salivary t-tau (right) and p-tau (left) levels in AD patients, PD patients, and healthy individuals. Data are presented as mean ± SEM. Figure B. The gel picture shows the amplified PCR product. The size of the PCR product was found to be at the expected size. Line 1: 1-kb marker, Lane 2: S2 cells, Lane 3: stable cells expressing Gr5a. Figure C. Change of currents for AD, PD, and control groups using the EG-ISFET biosensor. The change of current generated from the sensing cells after it was normalized to the values obtained from control cells. Data are presented as median. Figure D. Change of currents for sensing and control cells using EG-ISFET biosensor. The change of current generated from the sensing and control cells from saliva samples. Data are presented as median. (ZIP) [file pone.0117810.s001.zip › Figure D.tif.tif]

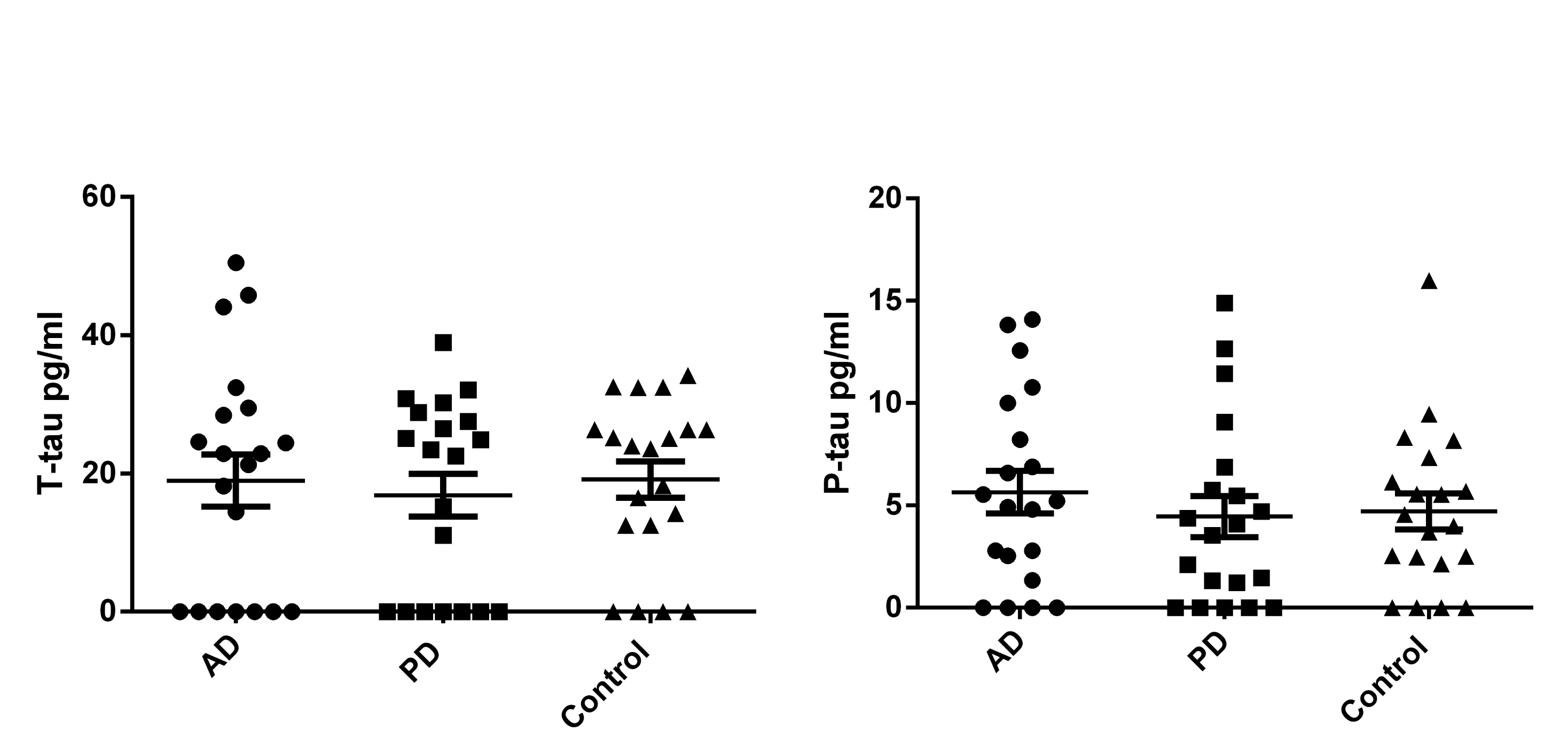

Supplement: S1 File — Figure A. Salivary AD biomarkers for three different groups. Salivary t-tau (right) and p-tau (left) levels in AD patients, PD patients, and healthy individuals. Data are presented as mean ± SEM. Figure B. The gel picture shows the amplified PCR product. The size of the PCR product was found to be at the expected size. Line 1: 1-kb marker, Lane 2: S2 cells, Lane 3: stable cells expressing Gr5a. Figure C. Change of currents for AD, PD, and control groups using the EG-ISFET biosensor. The change of current generated from the sensing cells after it was normalized to the values obtained from control cells. Data are presented as median. Figure D. Change of currents for sensing and control cells using EG-ISFET biosensor. The change of current generated from the sensing and control cells from saliva samples. Data are presented as median. (ZIP) [file pone.0117810.s001.zip › Figure A.tif.tif]

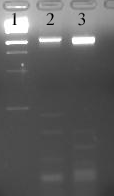

Supplement: S1 File — Figure A. Salivary AD biomarkers for three different groups. Salivary t-tau (right) and p-tau (left) levels in AD patients, PD patients, and healthy individuals. Data are presented as mean ± SEM. Figure B. The gel picture shows the amplified PCR product. The size of the PCR product was found to be at the expected size. Line 1: 1-kb marker, Lane 2: S2 cells, Lane 3: stable cells expressing Gr5a. Figure C. Change of currents for AD, PD, and control groups using the EG-ISFET biosensor. The change of current generated from the sensing cells after it was normalized to the values obtained from control cells. Data are presented as median. Figure D. Change of currents for sensing and control cells using EG-ISFET biosensor. The change of current generated from the sensing and control cells from saliva samples. Data are presented as median. (ZIP) [file pone.0117810.s001.zip › Figure B.tif.tif]

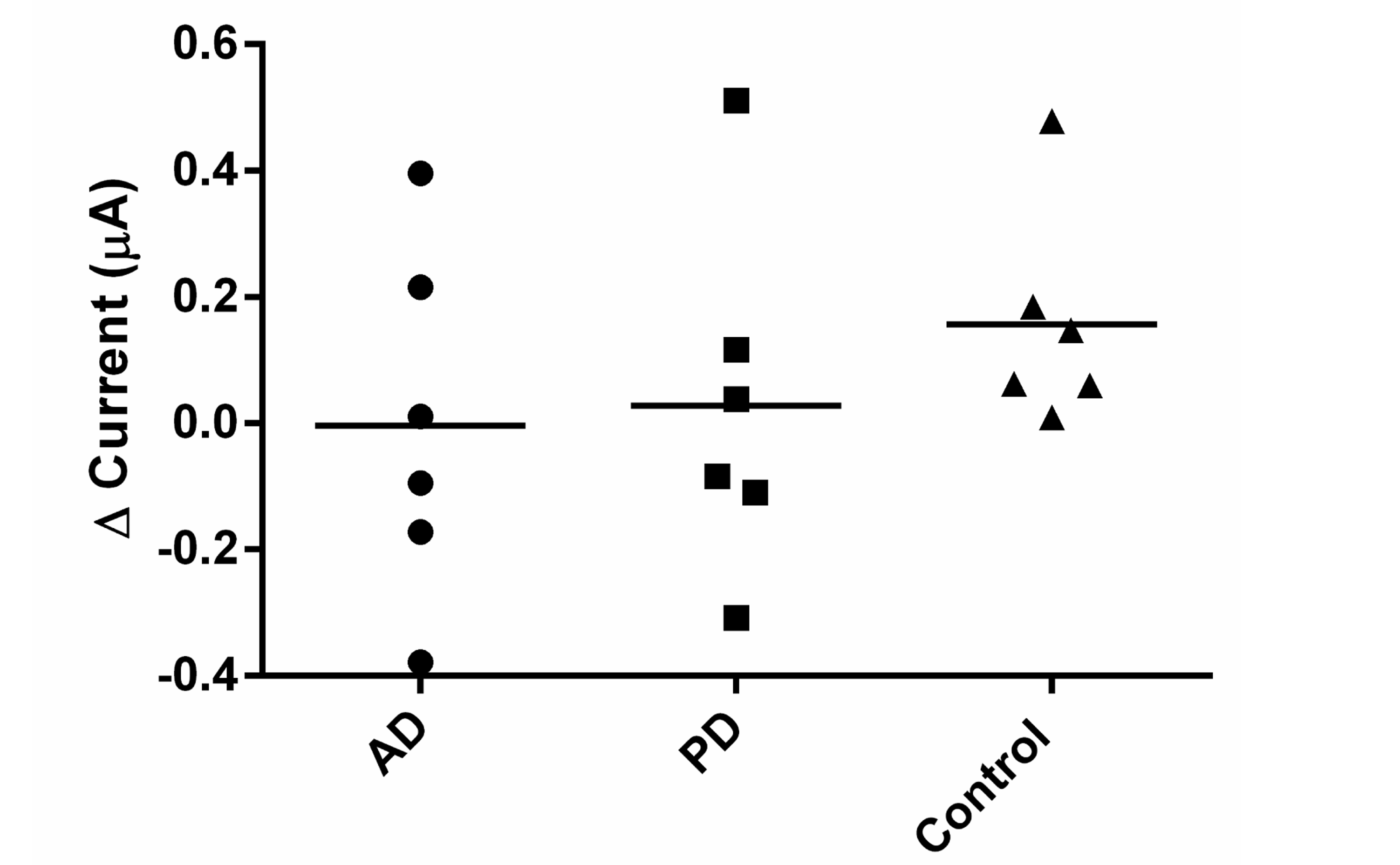

Supplement: S1 File — Figure A. Salivary AD biomarkers for three different groups. Salivary t-tau (right) and p-tau (left) levels in AD patients, PD patients, and healthy individuals. Data are presented as mean ± SEM. Figure B. The gel picture shows the amplified PCR product. The size of the PCR product was found to be at the expected size. Line 1: 1-kb marker, Lane 2: S2 cells, Lane 3: stable cells expressing Gr5a. Figure C. Change of currents for AD, PD, and control groups using the EG-ISFET biosensor. The change of current generated from the sensing cells after it was normalized to the values obtained from control cells. Data are presented as median. Figure D. Change of currents for sensing and control cells using EG-ISFET biosensor. The change of current generated from the sensing and control cells from saliva samples. Data are presented as median. (ZIP) [file pone.0117810.s001.zip › Figure C.tif.tif]
